# Atopic manifestations of inborn errors of immunity

**DOI:** 10.1097/ACI.0000000000000943

**Published:** 2023-09-26

**Authors:** Laura Sams, Sonali Wijetilleka, Mark Ponsford, Andrew Gennery, Stephen Jolles

**Affiliations:** a Paediatric Haematopoietic Stem Cell Transplant Unit, Great North Children's Hospital (GNCH), Royal Victoria Infirmary, Queen Victoria Road; b Translational and Clinical Research Institute, Faculty of Medical Sciences, Newcastle University, Newcastle upon Tyne; c Immunodeficiency Centre for Wales, University Hospital of Wales, Cardiff, UK

**Keywords:** atopy, eosinophilia, hyper IgE, inborn errors of immunity, STAT6 Gain of function

## Abstract

**Purpose of review:**

Allergy and atopic features are now well recognized manifestations of many inborn errors of immunity (IEI), and indeed may be the hallmark in some, such as DOCK8 deficiency. In this review, we describe the current IEI associated with atopy, using a comprehensive literature search and updates from the IUIS highlighting clinical clues for underlying IEI such as very early onset of atopic disease or treatment resistance to enable early and accurate genetic diagnosis.

**Recent findings:**

We focus on recently described genes, their categories of pathogenic mechanisms and the expanding range of potential therapies.

**Summary:**

We highlight in this review that patients with very early onset or treatment resistant atopic disorders should be investigated for an IEI, as targeted and effective therapies exist. Early and accurate genetic diagnosis is crucial in this cohort to reduce the burden of disease and mortality.

## INTRODUCTION

Inborn errors of immunity (IEI) associated with atopy provide valuable insights into the pathophysiology of the immune system and pathways responsible for atopic disease. Atopy is a recognized component of a growing number of IEI as wider phenotypes are defined, and in some may be the predominant manifestation. Given atopy-related manifestations of these diseases may present to a range of clinical specialists across infancy to adulthood, we set out to summarise recent developments in this field. We highlight novel genetic conditions that may present at this interface, including gain of function mutations in the IKAROS transcription factor [1^▪▪^], and autosomal dominant gain of functions (GOF) in signal transducer and activator of transcription 6 (STAT6) [2^▪▪^,^3^]. Finally, we propose an updated mechanistic framework for the development of atopy at the interface of IEI whilst highlighting pitfalls for associated complications, and opportunities for precision therapy. 

**Box 1:**
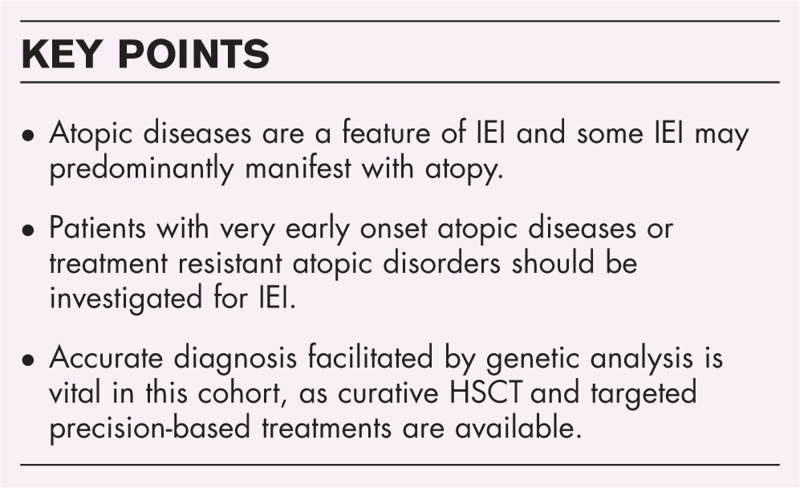
no caption available

## MATERIALS AND METHODS

**FIGURE 1:**
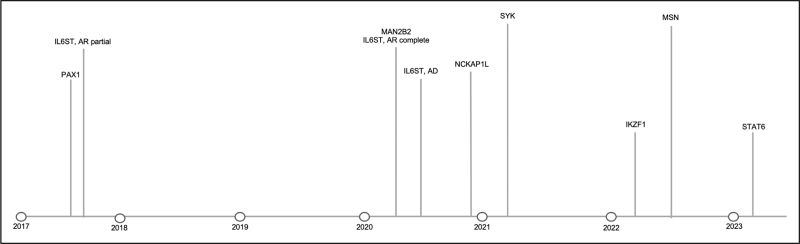
Updated timeline of genes discovered responsible for inborn errors of immunity associated with atopy [1^▪▪^,^2^^▪▪^,4^▪^,^17^,^19^–^27^].

We conducted a rapid literature review using the search terms ‘Atopy AND primary immunodeficiency’ OR ‘atopy’ AND ‘inborn errors of immunity’ in PubMed, considering articles published between 1 January 2020 and 1 June 2023. Particular attention was given to monogenic disorders added to the International Union of Immunological Societies (IUIS) 2022 update of IEI [4^▪^]. We included disorders where case reports described clinically significant features of allergic rhinitis, asthma and atopic dermatitis (eczema), elevations in IgE or hypereosinophilia. Two independent reviewers classified each monogenic disorder within the predominant category of mechanism. Where disagreement arose regarding classification, a consensus was agreed with the wider team. We identified new genes with reported atopic presentations within the most recent 2022 IUIS IEI update combined with a rapid literature review. The dates these disorders were reported is shown in Fig. 1, and clinical phenotypes summarized in Table 1, adapted from Lyons *et al*. [5], and Nelson *et al*. [6^▪^]. Table 2 illustrates a comprehensive overview of all IEI associated with atopy, adapted from Lyons *et al*. [5], Nelson *et al*. [6^▪^] and IUIS update [4^▪^].

**Table 1:** Novel IEIs with atopic manifestations – diagnostic features, atopic prevalence and clinical pitfalls

	References	Total cases described	Age of diagnosis (years)	Key features	Atopic features	Clinical pitfalls
STAT6 GOF	Sharma *et al.*[2^▪▪^]	16	3–60	Early-onset atopy within the first year of life Treatment resistant atopy Recurrent viral infections Recurrent skin and respiratory infections	Eczema Food allergies Asthma Eosinophilic gastrointestinal (GI) disease Anaphylaxis Eosinophilia Elevated IgE	Lymphoma risk
RLTPR	Wang *et al.*[7]: six patients Shober [8]: four patients Yonkof [9]: two patients Sorte [10]: four patients Maccari [11]: one patient Anas M Alazami *et al.*[12]: seven patients Kurolap *et al.*[13]: one patient Magg *et al.*[14]: five patients Atschekzei [15]: three patients	Greater than 10	Not reported	Combined immunodeficiency (CID) Recurrent bacterial fungal and mycobacterial infections Skin infections e.g. molluscum, diffuse warts from Human papillomavirus (HPV) infection and abscesses Respiratory tract infections	Eczema Eosinophilic oesophagitis High IgE Asthma Food allergy Cold urticaria	Epstein–Barr virus (EBV) lymphoproliferation
IKZF1	Hoshino *et al.*[1^▪▪^]	8	over 40	Autoimmunity (diabetes, colitis, thyroiditis) Lymphoproliferation Plasma cell expansion Evans Syndrome Recurrent infections, Immune dysregulation	Food allergy Asthma Rhinitis Dermatitis Eosinophilic oesophagitis	IgG4-related disease (3/8)
NCKAP1L LOF	Cook *et al.*[16] Castro *et al.*[17]	9	15 months – 11 years	Autoinflammatory Recurrent upper respiratory tract infection (URTI) Skin abscesses	Eczema Elevated IgE	
MSN	Lagresle-Peyrou *et al.*[18] and Fang *et al.*[19]	16	Not reported	Recurrent infections with bacteria and varicella and molluscum contagiosum Neutropenia Decreasing immunoglobulin over time	Eczema Atopic dermatitis	Very Early Onset Inflammatory Bowel Disease (VEOIBD) (1 case report)

**Table 2:** Atopy as a manifestation of IEI

Mechanism of pathogenesis	Associated genes	Immunological features of presentation	Atopic features of presentation	Mode of inheritance
Impaired skin and mucosal barrier function	*FLG*	Skin infections	Atopic dermatitis Food allergy Allergic rhinitis Asthma Eosinophilia High IgE	Autosomal recessive
	SPINK5	Skin infections	Atopic dermatitis Food allergy Allergic rhinitis Asthma Eosinophilia High IgE	Autosomal recessive
	CDSN	Skin infections	Atopic dermatitis Food allergy Eosinophilia High IgE	Autosomal recessive
	*DSG1*	Skin infections	Atopic dermatitis Food allergy Eosinophilia High IgE	Autosomal recessive
	DSP	Skin infections	Atopic dermatitis Food allergy Eosinophilia High IgE	Autosomal recessive
	sIgA deficiency	Antibody deficiency Bacterial infections Autoimmunity	Asthma, food allergy, allergic rhinitis and eczema	Unknown
	NEMO	Monocyte dysfunction Low immunoglobulins	Atopic dermatitis Asthma Food allergies Allergic rhinitis	X-linked
Cytoskeletal abnormalities	WAS	CID	Atopic dermatitis Food allergy Eosinophilia High IgE	X-linked
	WIP	CID	Atopic dermatitis Food allergy Eosinophilia High IgE	Autosomal recessive
	DOCK8	CID Susceptibility to viral infections	Atopic dermatitis Food allergy Eosinophilia High IgE	Autosomal recessive
	STK4	CID	Atopic dermatitis Food allergy Eosinophilia High IgE	Autosomal recessive
	NCKAP1L deficiency	Autoinflammatory Recurrent URTI Skin abscesses	Atopic dermatitis	Autosomal recessive LOF
	ARPC1B	CID Recurrent invasive infections	Eosinophilia High IgE	Autosomal recessive
	MSN Less than 10 reported cases to date	Recurrent infections with bacteria and varicella Neutropenia Decreasing immunoglobulin over time	Atopic dermatitis	X-linked
Aberrant TCR signalling	CARD11	CID/SCID	Eosinophilia High IgE	Autosomal recessive
	CARD11	Cutaneous viral infections Recurrent respiratory tract infections	Atopy Eosinophilia	Autosomal dominant LOF (dominant negative)
	BCL10	CID/SCID	Eosinophilia High IgE	Autosomal recessive
	MALT1	CID/SCID	Eosinophilia High IgE	Autosomal recessive
	CARML2	CID	Eosinophilia High IgE	Autosomal recessive
	ZAP70	CID/SCID	Eosinophilia High IgE	Autosomal recessive
	LAT	CID/SCID	Eosinophilia High IgE	Autosomal recessive
	RLTPR deficiency	CID Recurrent bacterial, fungal and mycobacterial infections Skin infections e.g. molluscum, diffuse warts from HPV infection, and abscesses Respiratory tract infections EBV lymphoproliferation	Atopic dermatitis Eosinophilic oesophagitis High IgE Asthma Food allergy Cold urticaria	Autosomal recessive
Disrupted cytokine signalling	IL6RA	Skin infections Respiratory tract infections Recurrent pyogenic infections Abscesses	Atopic dermatitis Eosinophilia High IgE	Autosomal recessive
	IL6ST	Skin infections Respiratory tract infections Bronchiectasis Boils Aspergillosis	Atopic dermatitis Eosinophilia High IgE	Autosomal recessive/autosomal dominant
	STAT3	Skin infections Respiratory tract infections	Atopic dermatitis Eosinophilia High IgE	Autosomal dominant
	ZNF341	Skin infections Respiratory tract infections	Atopic dermatitis Eosinophilia High IgE	Autosomal recessive
	IL21R Less than 10 reported cases to date	CID Recurrent infections including PCP and cryptosporidium	Increased IgE	Autosomal recessive
	TGFBR1/2 (Loeys – Dietz syndrome)	CID Recurrent respiratory tract infections	Eczema Food allergies	Autosomal dominant
	ERBB21P (ERBIN deficiency) One case/kindred been reported to date	CID Recurrent respiratory tract infections Susceptibility to Staph aureus	Atopic dermatitis Moderately increased IgE	Autosomal dominant
	STAT5B	CID Hypergammaglobulinaemia Autoimmunity	Atopic dermatitis High IgE	Autosomal recessive/autosomal dominant
	STAT5B GOF	Normal immunoglobulin levels, T cells and B cells Diarrhoea	Atopic dermatitis Urticaria Eosinophilia Hypereosinophilic syndrome	Unknown
	PIK3CG Less than 10 reported cases to date	Antibody deficiency Recurrent infections	Eosinophilia	Autosomal recessive
	JAK1 (GOF) One case/kindred been reported to date	Immune dysregulation Autoimmunity Viral infections	Eosinophilic enteritis Eosinophilia	Autosomal dominant
	TYK2	Susceptibility to viruses Multiple cytokine signalling defects	Elevated IgE	Autosomal recessive
	OTULIN Less than 10 reported cases to date	Autoinflammatory Neonatal recurrent fever Neutrophilia	Dermatitis	Autosomal recessive
	SYK Less than 10 reported cases to date	Autoinflammatory Recurrent infections Multiorgan inflammatory disease Dysgammaglobulinaemia B-cell lymphoma	Dermatitis	Autosomal dominant GOF
Regulatory T cell Disorders	FOXP3	Autoimmunity	Atopic dermatitis Food allergy Asthma Eosinophilia High IgE	X-linked
	IL2RA	CID Autoimmunity	Atopic dermatitis Food allergy Asthma Eosinophilia High IgE	Autosomal recessive
	IKZF1 Less than 10 reported cases to date	Autoimmunity Recurrent infections	Allergy	Autosomal dominant GOF
	IL2RB (CD122 deficiency) 5 kindreds	Immune dysregulation Autoimmunity Autoimmune haemolytic anaemia Hypergamma Viral infections – EBV, CMV	Dermatitis	Autosomal recessive
Innate cell effector mechanisms	PLCG2	CVID Autoimmunity Autoinflammatory	Temperature-sensitive mast cell degranulation	Autosomal dominant
	NLRP3	Autoinflammatory Fever Leukocytosis Conjunctivitis	Urticaria	Autosomal dominant GOF
Thymic development disorders	PAX1 Less than 10 reported cases to date	SCID Omenn's-like syndrome Severe, recurrent infections Athymic	Erythroderma Eosinophilia Normal to raised IgE	Autosomal recessive
	EXTL3 Less than 10 reported cases to date	CID Low Immunoglobulins	Eosinophilia	Autosomal recessive
	FOXN1	CID Recurrent viral and bacterial respiratory tract infections	Atopic dermatitis	Autosomal dominant
	22q11 deletion syndrome	CID Normal or decreased immunoglobulins May have low TRECs at newborn screening	Eczema Asthma	Autosomal dominant
Decreased T cell repertoire diversity	Multiple genes presenting as Omenn syndrome, such as RAG1/2, ADA, LIG4, ZAP70, etc.	Leaky SCID	Erythroderma Eosinophilia High IgE	Autosomal recessive
	BCL11B	CID	Severe atopic dermatitis Food allergies Allergic asthma Urticaria Eosinophilia Elevated IgE	Autosomal dominant
Metabolic	MAN2B2 One case/kindred reported to date	CID Recurrent infections	High IgE	Autosomal recessive
	PGM3	CID Recurrent pneumonia Recurrent skin abscesses Bacterial and viral infections	Severe atopy High IgE Eosinophilia	Autosomal recessive
	PEPD (prolidase deficiency)	Immune dysregulation Autoimmunity Autoantibodies Chronic skin ulcers Infections	Atopic dermatitis	Autosomal recessive

Genes are ordered into their associated pathway/mechanism of disease; however, there may be overlap between mechanisms of pathogenesis for the same gene (Adapted from Milner 2018, and Nelson 2022 and including recent IUIS updates). *FLG* and *DSG1* are marked in italics, as they are not necessarily associated with IEI but are monogenic defects supporting the pathogenic category. We have grouped thymic development disorders and decreased T cell repertoire diversity [4^▪^,^5^,6^▪^,^26^,^28^–^41^].

## CATEGORISATION OF MONOGENIC INBORN ERRORS OF IMMUNITY INTO MECHANISTIC PATHWAYS TO ATOPY

**FIGURE 2:**
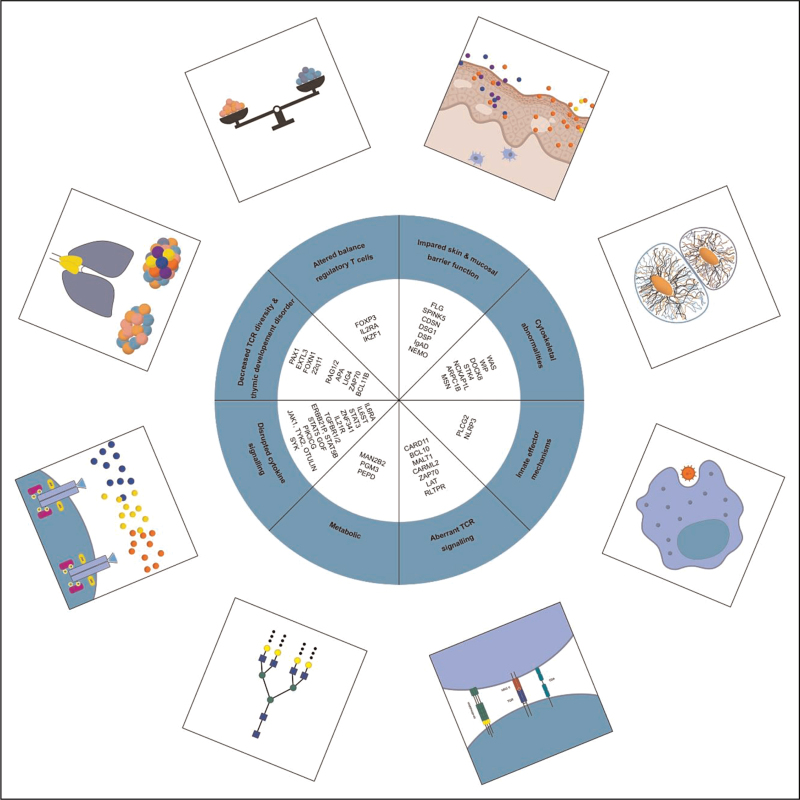
Categories of pathogenic mechanisms of atopy in inborn error of immunity.

Lyons *et al.*[5] proposed seven broad categories of inborn errors of immunity favouring development of atopy. Our adaptation has been modified to include the following eight categories, summarised in Fig. 2: impaired skin and mucosal barrier function; cytoskeletal abnormalities; aberrant TCR signalling; disrupted cytokine signalling; decreased T cell repertoire diversity and thymic development disorders; regulatory T cell (Treg) disorders; innate cell effector mechanisms; and metabolic disorders.

We describe an expansion in both the number of IEI with associated atopic manifestations and in the mechanistic categories underpinning the pathogenesis. This highlights the importance of awareness and early recognition of atopy as a manifestation of a growing number of IEI.

### Cytoskeletal abnormalities

Cytoskeletal disorders include Wiskott-Aldrich syndrome (WAS), Wiskott-Aldrich syndrome protein (WASP) WAP interacting protein (WIP), Dedicator of cytokinesis 8 (DOCK8) deficiency and Serine/threonine kinase 4 (STK4) deficiency. These cause a combined immunodeficiency with atopic features. WAS, DOCK8 and STK4 are also linked to a higher rate of autoimmunity and malignancy, illustrating the broad effects of immune dysregulation in IEI [4^▪^,^5^,6^▪^]. Deficiencies in the Nck-associated protein 1-like (*NCKAP1L*) gene, also known as hematopoietic protein 1 (HEM1), were first reported in humans by Castro *et al.*[17]. The gene encodes a haematopoietic lineage specific regulator of the actin cytoskeleton, vital for downstream signalling of activated Rac to stimulate F-actin polymerization in response to engagement of immune receptors [B cell receptor, TCR, Toll like receptor (TLR) and cytokine receptors] and is responsible for actin cytoskeleton reorganisation. Disruption to mechanistic Target of Rapamycin (mTOR) 2 and F-actin control results in immune dysregulation. Nine patients have been reported, for which a cohort of five patients from four unrelated families described by Cook *et al.*[16], had atopic and inflammatory diseases, chronic hepatosplenomegaly, lymphadenopathy with elevated IgE in 4 patients. Other features included recurrent bacterial and viral skin and respiratory infections and specific antibody deficiencies. Lymphoproliferation, cytokine overproduction, lymphadenopathy, hyperinflammation and autoimmune manifestations were also reported [17].

Variants in the MSN gene have recently been described as the cause of X-linked moesin-associated immunodeficiency (X-MAID). Sixteen cases have been reported worldwide. Patients with hemizygous mutations in the MSN gene present with lymphopenia, impaired T-cell proliferation, hypogammaglobulinemia, altered migration and adhesion capacities and susceptibility to bacterial and viral infections of the respiratory and gastrointestinal systems. Eight patients had skin manifestations mainly of eczema, molluscum contagiosum and atopic dermatitis [18,19]. MSN, ezrin and radixin are members of the ezrin-radixin-moesin (ERM) family which modulates the actin cytoskeleton and plasma membranes [42].

### Aberrant TCR signalling

Defective TCR signalling is evident in CARD11, BCL10, MALT1, CARML2, ZAP70, LAT and RLTPR deficiencies. Presentations consist of CID/severe combined immunodeficiency (SCID) with atopic features such as eosinophilia and high IgE. TCR signalling can either be absent or of reduced strength. Low strength signals between the TCR and major histocompatibility complex (MHC) complex have previously been demonstrated to skew naive T cell differentiation toward a T helper cell (Th) 2 response, promoting atopy [4^▪^,^5^,6^▪^]. Depending on the type of defect in CARD11, presentation can differ. Dominant negative mutations are associated with atopy, including moderate to severe dermatitis, high IgE and CID, like MALT1 deficiencies [5,6^▪^]. ZAP70 deficiency may manifest as atopic disease before the immunodeficiency becomes apparent [6^▪^].

RLTPR deficiency causes aberrant TCR signalling, by interfering with CD28 stimulation in T-cells [7]. Patients present with CID, recurrent bacterial, fungal and mycobacterial infections, and skin manifestations such as diffuse and recurrent warts. Atopic features include dermatitis, eosinophilic oesophagitis, asthma, food allergy, cold urticaria and high IgE [4^▪^,^5^,6^▪^].

### Disruption of cytokine signalling

Genetic defects causing ineffective cytokine signalling include *IL6RA*, *IL6ST*, *STAT3* and *ZNF341*.

Patients with dominant negative loss of function mutations in *STAT3*, present with recurrent infections, atopic dermatitis, eosinophilia, food allergy and high IgE. *ZNF341* is involved in *STAT3* gene expression and presents in a similar fashion. This condition promotes atopy, as *STAT3* phosphorylation leads to suppression of Th2 responses and favours Th17 responses, thereby reducing the propensity for atopy. Mutations in *STAT3* diminish this effect, resulting in increasing Th2 responses [6^▪^].

Autosomal dominant *STAT6* GOF variants associated with early-onset (<12 months) severe atopy have been reported by multiple groups [2^▪▪^,^3^,^43^–^45^]. Treatment-resistant atopic dermatitis and food allergies were most common, followed by asthma, eosinophilic gastrointestinal disease and anaphylaxis. Elevated IgE levels and eosinophilia were noted [2^▪▪^]. *STAT6* is an intracellular transcription factor downstream of IL4 and IL4R/JAK-kinase signalling cascade and a central node of immune polarization and a key modulator for the risk of allergic disease in humans and mice [3,46]. Translocation of *STAT6* to the nucleus, activates a pattern of gene expression mediating Th2 cell differentiation, M2 macrophage polarization, promotion of B cell survival and IgE class switching [47–50].

Seven kindreds were reported as sporadic, and three kindreds followed an autosomal dominant pattern of inheritance. Clinical features of wider immune dysregulation included recurrent nonfatal skin, respiratory, and viral infections identified in half of the cohort. Similar to characteristics of DN *STAT3* LOF, short stature, pathologic fractures and generalised hypermobility were described. One patient died due to anaphylaxis at aged 20 and the other aged 35 secondary to a cerebral aneurysm, demonstrating the severity of the multisystem disease in this cohort [2^▪▪^]. It is notable that somatic activating mutations in *STAT6* have been associated with B cell lymphoma [51–53]. The oldest patient in the cohort, experienced recurrent B cell lymphoma with follicular lymphoma aged 49 with subsequent relapse with a transformed follicular lymphoma (diffuse large B cell lymphoma) aged 60 [2^▪▪^].

### Decreased T cell repertoire diversity

This mechanism manifests as Omenn syndrome, a type of leaky SCID, associated with multiple genetic defects including recombination activating gene (RAG)1, RAG2 and adenosine deaminase (ADA). Hypomorphic mutations in the responsible genes result in a limited number of T cells which undergo oligoclonal expansion. These T cells preferentially differentiate into the Th2 lineage, causing the classical presenting symptoms of hepatosplenomegaly, lymphadenopathy, erythroderma, eosinophilia and high IgE [4^▪^,^5^,6^▪^].

Two hypotheses exist to explain how a reduced diversity of T cells can result in atopy. The first suggests that reduced T cell diversity causes a lack of Tregs and loss of regulation of Th2 with subsequent atopy. The second hypothesis suggests low strength TCR signalling leading to skewing of Th2 differentiation. Due to reduced thymopoiesis, there is a lack of T cells with high affinity receptors which leads to a preferential expansion of T cells with low affinity receptors that differentiate into Th2 cells, thus promoting atopy [5].

### Altered balance of conventional T cells and regulatory T cells

Reduced numbers of Tregs leads to a failure of tolerance and presents as autoimmunity and features of immune dysregulation such as atopy [5,6^▪^].

FOXP3 is the master transcription factor for Tregs, and its deficiency is responsible for immunodysregulation polyendocrinopathy enteropathy X-linked (IPEX) syndrome. IPEX presents as autoimmunity with severe atopic dermatitis, food allergy, asthma, eosinophilia and high IgE [4^▪^,^5^,6^▪^].

IL2RA loss of function mutations lead to atopic features such as dermatitis, elevated IgE with autoimmunity and immunodeficiency. Tregs express the most IL2RA and fail to survive in its absence. IL-2 signalling through its receptor on Tregs promotes production of IL-10, promoting tolerance. Deficiencies in IL2RA result in loss of survival signals for Tregs and loss of suppressive function, favouring atopy [5,6^▪^].

### IKAROS gain-of-function mutations

Germline heterozygous IKAROS GOF mutations presented with profound autoimmunity and immune dysregulation (75%, 6/8) with an age of onset of less than 1 to over 40 years. The regulation of *IKZF1* is required for T helper cell, Treg and plasma cell differentiation [1^▪▪^].

Patients developed autoimmune diseases including type 1 diabetes mellitus, enteritis, autoimmune hepatitis, Hashimoto thyroiditis, leukocytoclastic vasculitis, vitiligo and alopecia with autoantibodies. GOF patients showed an absence of effector Treg and increased T follicular cell population, suggesting T-cell differentiation is compromised by abnormal IL-2 production. Autoimmune manifestations may be due to abnormal IL-2 production and effector Treg populations in these patients, as with other IEI patients with impaired Treg numbers and/or function IPEX syndrome and cytotoxic T-lymphocyte antigen 4 (CTLA-4) haploinsufficiency [54]. T cells expressing GOF mutations showed increased IL-4 (Th2) production, and decreased IL-2 and IFNγ production (Th1) [1^▪▪^,^55^].

Features also included atopy, lymphoproliferation and generally nonsevere bacterial infections. Whole-exome sequencing identified two patients with apparent autosomal dominant inheritance, as well as de novo occurrences. One patient harbouring a GOF mutation did not present with any clinical manifestations, demonstrating variable immunological penetrance.

Patients had mostly normal B-cell numbers, with normal to elevated immunoglobulin and IgE levels. Presentations of atopic disease included asthma, rhinitis, dermatitis, food allergy and eosinophilic oesophagitis. These are postulated to be due to increased Th2 differentiation with increased eosinophils, and production of IL-4 [56]. Increased IL-4 may result in Th2 and T follicular helper cell (TFH)2 skewing through negative regulation by IL-2 and/or hyper-IgE likely contributes to the development of allergic manifestations. Plasma cell hyper-proliferation was reported. Three patients had IgG4-related diseases demonstrated by an increased infiltration of the IgG4-positive plasma cells in the lymph nodes, intestine or bile duct [1^▪▪^].

### Skin barrier defects

Multiple genes are associated with disrupted skin barrier function and infection, summarized in Table 2.

The ‘atopic march’ is characterized by early onset eczema predisposing to developing allergic rhinitis, then subsequently asthma and food allergies [6^▪^]. It is suggested that increased skin permeability from eczema, leads to cutaneous antigen-presenting cells (APCs) being exposed to increased amounts of usually innocuous environmental antigens. This leads to sensitisation, and production of Th2 associated pro-inflammatory cytokines, consequently initiating the allergic response [5,6^▪^]. Skin barrier disruption alongside downregulation of protective antimicrobial peptides, increases infection risk [6^▪^].

Pro-inflammatory type 2 cytokines also downregulate filaggrin, an important protein for skin barrier integrity [57], due to its role in producing natural moisturising factor, essential for hydration, during normal skin desquamation [58]. Therefore, disturbances in filaggrin production result in dry, flaky skin, increasing skin permeability, allowing increased exposure to antigens, and so the cycle continues [5]. This is observed in ichthyosis vulgaris, due to a homozygous LOF mutation in filaggrin, resulting in early onset (first months of life) severe atopy with elevated IgE [5,6^▪^].

### Selective IgA deficiency

Selective IgA deficiency (sIgAD) has similarly been postulated to result in impaired mucosal barrier function resulting in greater sensitisation and propagation of allergy. Up to 40% of sIgAD patients have allergy as a presenting or only symptom [37,38], with up to 84% of patients having some form of allergic manifestation, asthma being the commonest [35], others include allergic rhinitis, eczema and food allergy [35].

### Ectodermal dysplasia and NF-κB essential modulator

Atopic features have been described in ectodermal dysplasia, including scalp dermatitis, atopic dermatitis and elevated IgE with positive skin prick tests [59].

Children with ectodermal dysplasia syndromes experience atopic symptoms more frequently compared to the general paediatric population, including asthma, food allergies, allergic rhinitis and eczema [40] due to skin barrier disruption [60] and hypohidrosis or anhidrosis, fuelling their atopic march [61].

NEMO deficiency is associated with eczema and erythroderma [62].

### Thymic development disorders

Atopy in chromosome 22q11.2 deletion syndrome (22q11.2del) is proposed to be related to T-cell lymphopenia and homeostatic pressure driving Th2 polarization [63]. Atopy has been associated with low T-cell receptor excision circles, with low T cells conferring nearly a three-fold increased risk of allergy [64,65], with patients presenting with asthma, rhinitis/conjunctivitis, food allergy and atopic dermatitis. Other IEI in this category are PAX1, EXTL3 and FOXN1.

### Metabolic disorders

Mutations in MAN2B2 and PGM3 are congenital disorders of glycosylation (CDGs) [22,66].

Biallelic mutations in MAN2B2 have been shown to result in a CID, characterised by recurrent pneumonia, thrush, chronic diarrhoea and elevated IgE. Extra-immunological manifestations included small vessel vasculitis and thrombotic stroke [4^▪^,^22^].

PGM3 deficiency is regarded as a HIES [4^▪^]. Patients suffer from recurrent bacterial and viral infections, commonly affecting the skin and respiratory tract, low T cells and reduced memory B cells. Autoimmunity, along with severe atopy, including severe atopic dermatitis, food allergies and asthma have been reported, accompanied by marked eosinophilia and high IgE. Extra-immunological manifestations include neurological impairment, such as sensorineural hearing loss, low IQ, developmental delay and facial dysmorphism [4^▪^,^66^].

## TREATMENT UPDATES – FOCUS ON PRECISION THERAPIES

Improvements in genetic analysis have facilitated early diagnosis and options for precision therapy to modulate these defects. An expanding range of biologics and small molecule drug inhibitors are available for asthma or eczema, such as Mepolizumab (anti-IL5), Dupilumab (anti-IL4Rα) and Tezepelumab (antithymic stromal lymphopoietin) with potential for translational repurposing to rare diseases.

Dupilumab has been shown to be well tolerated and effective in a number of atopic diseases, especially refractory eczema. The IL-4α receptor antagonist inhibits the IL-13/ IL-4/ STAT 6 axis, disrupting IL-4 signalling and the allergic type 2 cytokine signature [67].

Dupilumab was highly effective in the three patients with *STAT6* GOF variants, demonstrating clinical and immunological biomarker and cutaneous improvement with increased growth velocity and weaning or discontinued oral corticosteroids. Preclinical data have suggested that Janus kinase (JAK) inhibitors such as Tofacitinib and Ruxolitinib may be beneficial [2^▪▪^]. Phase II studies are ongoing with Bruton's tyrosine kinase inhibitors (BTKi) in atopic dermatitis [68].

Dupilumab used in autosomal dominant AD *STAT3* LOF showed improved atopic dermatitis, eosinophilic folliculitis and recurrent cutaneous infections [69]. Improvements to other manifestations such as asthma and allergic bronchopulmonary aspergillosis have been reported [70,71]. Dupilumab has also been used to successfully treat severe atopic dermatitis in a patient with CARD11-associated atopy with dominant interference of NF-kB signalling (CADINS) [72].

There are case and single-centre reports for the use of Omalizumab in IEI, such as in AD STAT3 LOF with concomitant respiratory manifestations; however, its role is still to be defined. Glutamine supplementation for dominant negative CARD11 variants has not yet translated to clinical therapy. Oral dietary supplementation is a research avenue for phosphoglucomutase 3 (PGM3) deficiency, with evidence suggesting *in-vitro* supplementation with the nondiabetogenic amino-sugar N-acetylglucosamine (GlcNAc) led to normalised intracellular UDP-GlcNAc, surface CTLA-4 expression and alterations in cellular glycosylation and immune pathways [66,73]. The use of lenalidomide has been shown to lead to degradation of IKZF1 and prevent some of the abnormal IKZF1 GOF using *in vitro* assays [1^▪▪^].

### Role and effectiveness of allergen immunotherapy

Primary immunodeficiencies are described as a relative contraindication to commencing AIT; however, no controlled studies have investigated the effectiveness or associated risks. AIT is likely to have been performed in many cases of undiagnosed selective IgA deficiency [74,75].

The current European Academy of Allergy & Clinical Immunology (EAACI) guidelines state that careful consideration, on a case-by-case basis, with discussion between patient and the treating physician is required before deciding whether or not to commence AIT [76]. The British Society for Allergy & Clinical Immunology (BSACI) guidelines for venom immunotherapy (VIT) also state the effects of VIT in patients with disorders of the immune system such as immunodeficiency are not known and therefore the decision to offer treatment should be based on an individual ‘risk-benefit’ analysis [77]. We support individual consideration of patients with IEI for AIT, accepting that the efficacy remains unclear.

### Haematopoietic stem cell transplantation

HSCT is curative for certain IEI and may lead to resolution of atopy, however the durability remains unknown. IgE levels substantially decreased post-HSCT in the majority of patients who underwent transplantation for DN *STAT3* LOF and *DOCK 8* deficiency [78–80] alongside resolution of eczema post-HSCT [78,79]. Allergen-specific IgE also declined post-HSCT in all patients tested with DOCK 8 deficiency. Al-Herz *et al.*[80] reported, in 10 patients (91%) with DOCK 8 deficiency who presented with food allergy and food allergen-specific IgE levels, that food allergies clinically resolved post-HSCT in eight out of 10 patients confirmed by oral challenges, although not all studies confirmed this [81].

## CONCLUSION

Presentations of atopy should be considered as part of an underlying IEI and would warrant investigation particularly if early onset, refractive to treatment and with concurrent signs of autoimmunity, lymphoproliferation and recurrent infections. Patients who remain undiagnosed have a higher risk of morbidity and mortality. An initial immunological assessment proceeding to genetic testing aids early identification of specific genetic abnormalities enabling precision treatments improving outcomes for atopic disease in IEI.

## Acknowledgements

*L.S. and S.W. are joint first authors.*

*A.G. and S.J. joint last authors.*

*The authors would like to thank Harriet Gibson for her expert development of the illustrations.*

### Financial support and sponsorship

*None.*

### Drug information

*Mepolizumab: GlaxoSmithKline, North Carolina and Pennsylvania, USA.*

*Tezepelumab: Astrazeneca, Delaware, USA.*

*Dupilumab: jointly by Regeneron New York USA and Sanofi Paris France.*

*Tofacitinib: National Institutes of Health Maryland USA and Pfizer New York, USA.*

*Ruxolitinib by Incyte Corp Delaware USA and by Novartis Basel Switzerland elsewhere in the world.*

*Lenalomide: Bristol Myers Squibb, New York, USA.*

### Conflicts of interest

*L.S., S.W., M.P., A.G. and S.J. have no conflicts of interest to declare.*

## REFERENCES AND RECOMMENDED READING

Papers of particular interest, published within the annual period of review, have been highlighted as:

▪ of special interest
▪▪ of outstanding interest
